# Supplementary material for: The interactome of intact mitochondria by cross-linking mass spectrometry provides evidence for coexisting respiratory supercomplexes
Source: Mol Cell Proteomics. 2017 Dec 8;17(2):216–32. doi: 10.1074/mcp.RA117.000470 (PMC5795388; doi:10.1074/mcp.RA117.000470)
Supplement: Supplemental Data [file supp_RA117.000470_133922_0_supp_23978_fzffwf.zip › spectra_annotation/mito_spectra_annotation/418-1-1-1-9-1.pdf]

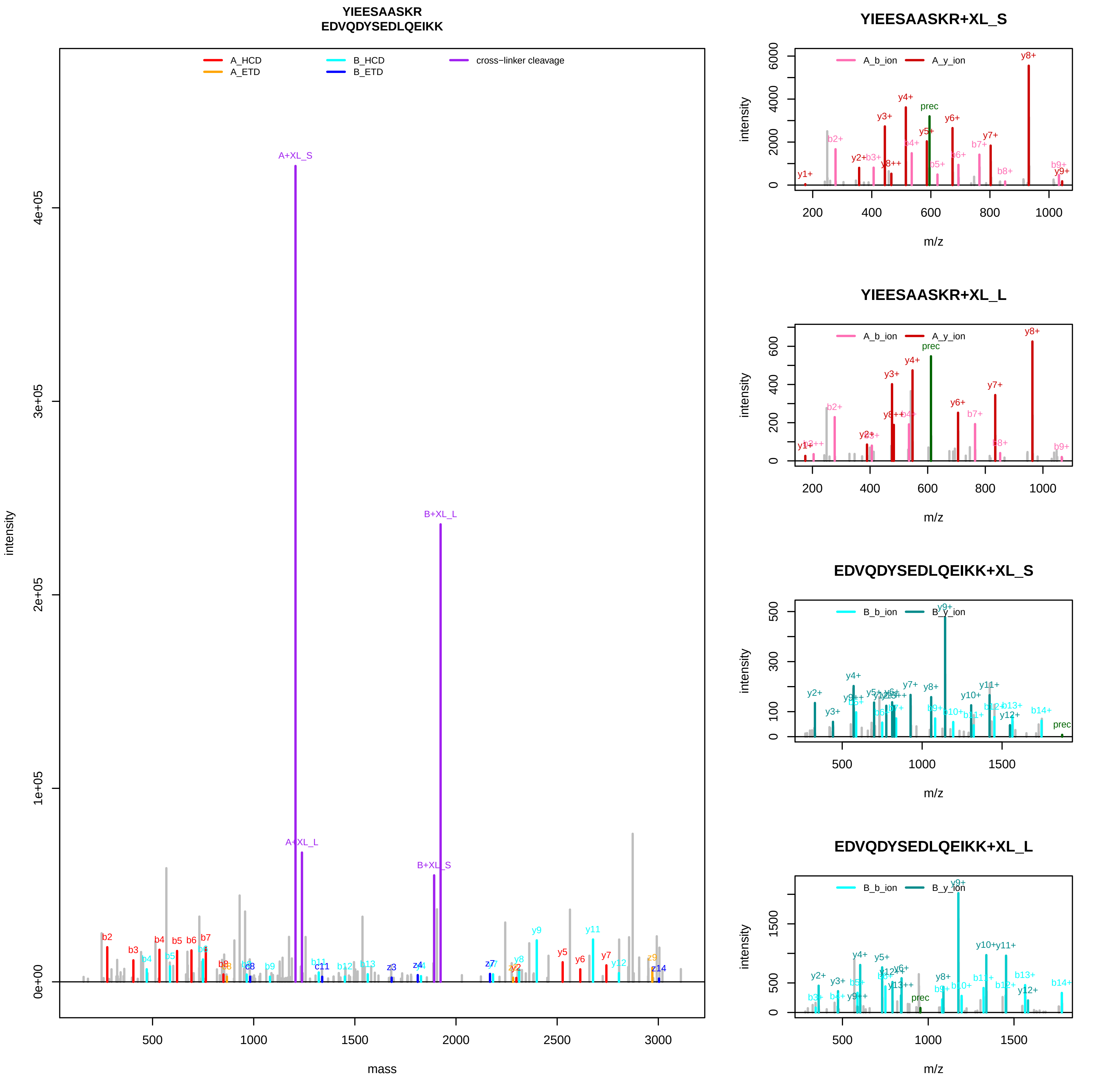
YIEESAASKR
EDVQDYSEDLQEIKK
A_HCD
A_ETD
B_HCD
B_ETD
cross−linker cleavage
A+XL_S
B+XL_L
A+XL_L
B+XL_S
intensity
mass
0e+00
1e+05
2e+05
3e+05
4e+05
500
1000
1500
2000
2500
3000
b2
b3
b4
b5
b6
b7
b8
c8
b9
c11
b11
b12
b13
z3
z4
z7
y8
y9
y11
y12
y5
y6
y7
z9
z14
YIEESAASKR+XL_S
YIEESAASKR+XL_L
EDVQDYSEDLQEIKK+XL_S
EDVQDYSEDLQEIKK+XL_L
A_b_ion
A_y_ion
B_b_ion
B_y_ion
prec
m/z
intensity
